# The Prevalence of Dementia in Korean American Older Adults in Maryland: The Memory and Aging Study of Koreans (MASK)

**DOI:** 10.1093/geroni/igaf122.363

**Published:** 2025-12-31

**Authors:** Hyun Kim, Patrick Walsh, Jean Guan, Hochang Lee

**Affiliations:** Columbia University, New York, New York, United States; University of Rochester, Rochester, New York, United States; University of Missouri School of Medicine, Springfield, Missouri, United States; University of Rochester, Rochester, New York, United States

## Abstract

Korean American (KA) older adults are among the fastest growing ethnic groups in the U.S. older adult population. KAs face an elevated risk of dementia due to high rates of well-established risk factors (e.g., cardiovascular and lifestyle factors). However, data on the prevalence of dementia in this population remain limited. To address this gap, Memory and Aging Study of Koreans (MASK) was developed, recruiting community-representative sample of KA older adults from ethnic churches, senior centers, and business establishments of Baltimore-Washington D.C. area. A total of 1,118 KA older adults were screened by trained, bilingual community health workers based on a Korean version of the Mini-Mental State Exam during the initial survey stage, and 91 participants underwent a second-stage clinical evaluation, including the Clinical Dementia Rating scale and physician evaluation. The estimated prevalence of dementia was 7.2% (SE ± 2.3), while mild cognitive impairment (MCI) was estimated to be 27.8% (SE = 5.9). Dementia prevalence increased with age (e.g., 35.3% in those aged 80+ years vs. 2.1% in those aged 70-79 years) and differed by subtypes (4.8% Alzheimer’s dementia vs. 2.4 vascular subtype). These findings provide a crucial first step in addressing dementia burden in culturally and linguistically isolated KA minority groups across the U.S.

